# Economic analysis of implementing virtual reality therapy for pain among hospitalized patients

**DOI:** 10.1038/s41746-018-0026-4

**Published:** 2018-06-13

**Authors:** Sean D. Delshad, Christopher V. Almario, Garth Fuller, Duong Luong, Brennan M. R. Spiegel

**Affiliations:** 1 Cedars-Sinai Center for Outcomes Research and Education (CS-CORE), Los Angeles, CA USA; 2 Department of Medicine, David Geffen School of Medicine at UCLA, Los Angeles, CA USA; 3 Division of Digestive and Liver Diseases, Cedars-Sinai Medical Center, Los Angeles, CA USA; 4 Division of Informatics, Cedars-Sinai Medical Center, Los Angeles, CA USA; 5 Department of Pharmacy, Cedars-Sinai Medical Center, Los Angeles, CA USA; 6 Department of Health Policy and Management, UCLA Fielding School of Public Health, Los Angeles, CA USA

**Keywords:** Health care economics, Pain

## Abstract

Virtual reality (VR) has emerged as a novel and effective non-pharmacologic therapy for pain, and there is growing interest to use VR in the acute hospital setting. We sought to explore the cost and effectiveness thresholds VR therapy must meet to be cost-saving as an inpatient pain management program. The result is a framework for hospital administrators to evaluate the return on investment of implementing inpatient VR programs of varying effectiveness and cost. Utilizing decision analysis software, we compared adjuvant VR therapy for pain management vs. usual care among hospitalized patients. In the VR strategy, we analyzed potential cost-savings from reductions in opioid utilization and hospital length of stay (LOS), as well as increased reimbursements from higher patient satisfaction as measured by the Hospital Consumer Assessment of Healthcare Providers and Systems (HCAHPS) survey. The average overall hospitalization cost-savings per patient for the VR program vs. usual care was $5.39 (95% confidence interval –$11.00 to $156.17). In a probabilistic sensitivity analysis across 1000 hypothetical hospitals of varying size and staffing, VR remained cost-saving in 89.2% of trials. The VR program was cost-saving so long as it reduced LOS by ≥14.6%; the model was not sensitive to differences in opioid use or HCAHPS. We conclude that inpatient VR therapy may be cost-saving for a hospital system primarily if it reduces LOS. In isolation, cost-savings from reductions in opioid utilization and increased HCAHPS-related reimbursements are not sufficient to overcome the costs of VR.

## Pain management: the real cost of virtual reality

Implementing virtual reality (VR) programs for inpatient pain management can potentially save hospitals money. Recent studies have highlighted VR as an effective alternative to traditional opioid treatments for the management of pain. Brennan Spiegel, at Cedars-Sinai Medical Center in Los Angeles, and colleagues carried out an economic analysis to determine the cost implications of implementing inpatient VR therapy programs for acute pain management in different US hospital settings. They found that such programs are cost-saving when they reduce patients’ length of stay in the hospital. However, the projected costs for inpatient VR programs are higher than the savings that can be made from decreases in opioid use or additional income from Hospital Consumer Assessment of Healthcare Providers and Systems-related reimbursements through increased patient satisfaction alone.

## Introduction

Effective pain management among hospitalized patients is associated with better health outcomes^1^ and increased patient satisfaction.^2^ Traditionally, pharmacologic therapies such as opioids form the cornerstone of pain management in the inpatient setting. However, while opioids are effective in reducing pain, they are also associated with side effects including sedation, dizziness, nausea, and constipation, among others. These adverse effects can prolong length of stay (LOS) in the hospital, increase healthcare costs, and decrease patient satisfaction.^3,4^

Non-pharmacologic therapies may contribute to the efficacy of an overall pain management strategy and provide alternatives to traditional opioid treatments.^5–7^ Recently, virtual reality (VR), a computer-generated simulation of a three-dimensional environment which can be explored and interacted with by the user, has emerged as a novel non-pharmacologic therapy for pain. There is an increasing body of evidence that demonstrates the effectiveness of VR on pain reduction in the outpatient setting.^8–12^ Aside from pain management, VR has also been tested in a variety of other disease states such as anxiety,^13–15^ obesity,^16–18^ oncology,^8^ and neurorehabilitation.^19,20^

Investigators, including those from our own group, have also examined the impact of VR on patients in the inpatient setting. In a feasibility study, we found that while few inpatients were both eligible and willing to use VR, those that used VR reported that it was a positive experience and that it improved their pain and anxiety.^21^ In a separate study, we found that 65% of hospitalized patients receiving a VR experience achieved a clinically significant pain response vs. 40% of controls watching a relaxation video (*p* = 0.01; number needed to treat = 4) without any adverse events reported.^22^ Given the effectiveness of VR therapy for pain management, VR as an adjunctive non-pharmacologic pain therapy program has potential to reduce opioid utilization.^23^ Other possible benefits of inpatient VR therapy include reduction in hospital LOS and increased patient satisfaction.^24,25^ While the use of VR in the hospital is promising, no study to our knowledge has yet examined the cost and effectiveness thresholds required for an inpatient VR program to be cost-saving.

To address this gap in knowledge, we sought to estimate the projected cost savings and budget impact of implementing a VR pain management program for hospitalized patients. We performed health economic decision analyses incorporating costs related to VR implementation, potential savings from decreases in opioid utilization and hospital LOS, and effects on Hospital Consumer Assessment of Healthcare Providers and Systems (HCAHPS) scores and resulting adjustments in Centers for Medicare & Medicaid Services (CMS) Hospital Value-Based Purchasing (VBP) payments. We then performed sensitivity analyses to create a return on investment (ROI) lookup table for hospitals of varying size and staffing costs that are considering implementation of an inpatient VR program. Our objective with this hypothesis-generating analysis was to create a framework for future health economic analyses of inpatient VR therapy and to determine the cost and effectiveness thresholds at which point inpatient VR therapy for pain management becomes cost-saving.

## Results

### Base-case results

Implementing a VR therapy program in the inpatient setting provided an average of $5.39 (95% confidence interval of –$11.00 to $156.17) in cost-savings per patient when compared to usual care. Among the sub-group of patients both eligible to receive and willing to use VR therapy (19.3% in the base-case), there was on average $98.49 savings per patient. For patients who did not receive VR therapy (80.7% of patients were not eligible or did not accept) the hospital lost $16.90 per patient.

### Tornado analysis results

**Fig. 1:**
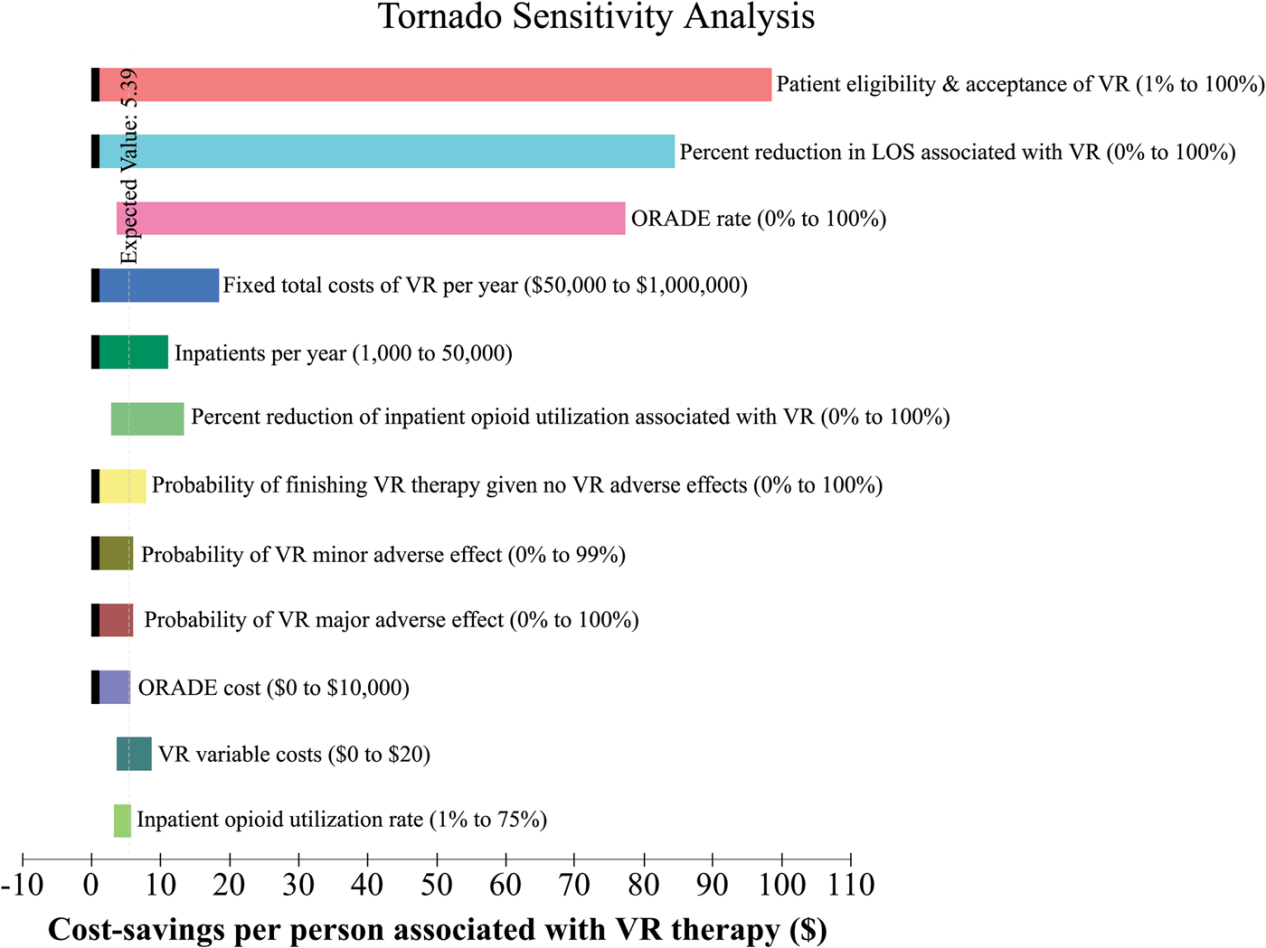
Tornado Analysis. Results of the multiple univariate sensitivity analysis testing the influence of variables on the model. Each bar demonstrates the range of cost-savings potential associated with the range (described in parentheses) tested for various variables (described in text next to each bar). A black indentation on the left-side of a bar indicates that the lowest value of cost-savings associated with the variable over the range tested is zero. The cost savings potential of the VR therapy program was most dependent on patient acceptance and eligibility and percent reduction in patient length of stay. Other significant factors include ORADE rate, costs of VR, number of admissions per year, and the degree to which VR therapy decreases opioid utilization. The probability of patients selecting the best possible answers on the HCAHPS survey had the least impact on the outcome of the model, and was not included in this figure. VR, virtual reality; ORADE, opioid-related adverse drug event; HCAHPS, Hospital Consumer Assessment of Healthcare Providers and Systems

Tornado analysis results are presented in Fig. 1. Patient acceptance and eligibility of VR and the reduction in length of stay associated with VR had the largest impact on the cost-savings potential of implementing VR therapy in the hospital setting. Other significant factors include the degree to which VR therapy decreases opioid utilization, ORADE rate, number of admissions per year, and the costs of VR therapy implementation. The probability of patients selecting the best possible answers on the HCAHPS survey had the least impact on the outcome of the model.

### Base-case sensitivity analyses

Because the base-case assumptions of the model may not be reproducible across hospitals, we performed sensitivity analysis to test the model using other probability and cost estimates (Table 1). One-way sensitivity analysis revealed that the VR program would remain cost-saving so long as the following are true: (1) at least 14.6% of patients utilize VR therapy; (2) total fixed costs of the VR program are less than $326,872; (3) VR variable costs are less than $31.27; (4) the probability of a VR minor or major adverse effect is less than 21.8% or 0.06%, respectively; (5) there are at least 11,485 admissions per year; and (6) VR reduces the marginal costs of the last day of hospitalization by at least 14.6%. In the base-case, VR remained cost-saving despite variation in opioid-related variables such as the cost of an ORADE, the probability of an ORADE, or the decrease in opioid utilization associated with a VR therapy program.

**Table 1:** Results of one-way sensitivity analyses

Variable	Base-case estimate	Threshold	Comment
VR-Related			
Probability of patient eligibility and acceptance of VR therapy	19.3%	14.6%	If percentage exceeds this threshold, VR therapy is cost-saving
Total yearly fixed costs of VR program	$246,090	$326,872	If cost remains below this threshold, VR therapy is cost-saving
VR variable cost	$3	$31	If cost remains below this threshold, VR therapy is cost-saving
Probability of minor VR adverse effects	2.5%	21.8%	If probability remains below this threshold, VR therapy is cost-saving
Probability of a major VR adverse effects	0.025%	0.06%	If probability remains below this threshold, VR therapy is cost-saving
Probability of finishing VR therapy given no adverse effects	90.0%	68.7%	If probability exceeds this threshold, VR therapy is cost-saving
Opioid-Related			
Percentage decrease in inpatient opioid utilization secondary to VR therapy	24.0%	NA	VR therapy is cost-saving even if it does not affect opioid utilization rate
Probability of an ORADE	2.4%	NA	VR therapy is cost-saving even if the ORADE rate is 0
Cost of an ORADE	$3,457	NA	VR therapy is cost-saving regardless of the cost of an ORADE
Hospital-related			
Hospital admissions per year	15,000	11,485	If number exceeds this threshold, VR therapy is cost-saving
Marginal cost associated with final day of hospitalization	$584	$425	If cost exceeds this threshold, VR therapy is cost-saving
Percentage of marginal cost savings of final day of hospitalization for patients receiving VR therapy without adverse effects	20%	14.6%	If percentage exceeds this threshold, VR therapy is cost-saving

*VR* virtual reality, *ORADE* opioid-related adverse drug event, *NA* not applicable

In a Monte Carlo probabilistic sensitivity analysis with 1000 simulations testing ranges described in Table 2, VR therapy remained cost-saving in 89.2% of head-to-head trials. The model was highly sensitive to the number of patients served by the VR program and its total costs. Table 3 provides an ROI lookup table based on hospital admissions per year and total annual fixed costs of a VR therapy program.

**Table 2:** Return on investment lookup table

	Total fixed costs of VR Program per year										
		$100,000	$200,000	$300,000	$400,000	$500,000	$600,000	$700,000	$800,000	$900,000	$1,000,000
Admissions per Year	**5000**	1.79	(18.21)	(38.21)	(58.21)	(78.21)	(98.21)	(118.21)	(138.21)	(158.21)	(178.21)
	**10,000**	11.79	1.79	(8.21)	(18.21)	(28.21)	(38.21)	(48.21)	(58.21)	(68.21)	(78.21)
	**15,000**	15.13	8.46	1.79	(4.88)	(11.54)	(18.21)	(24.88)	(31.54)	(38.21)	(44.88)
	**20,000**	16.79	11.79	6.79	1.79	(3.21)	(8.21)	(13.21)	(18.21)	(23.21)	(28.21)
	**25,000**	17.79	13.79	9.79	5.79	1.79	(2.21)	(6.21)	(10.21)	(14.21)	(18.21)
	**30,000**	18.46	15.13	11.79	8.46	5.13	1.79	(1.54)	(4.88)	(8.21)	(11.54)
	**35,000**	18.93	16.08	13.22	10.36	7.51	4.65	1.79	(1.07)	(3.92)	(6.78)
	**40,000**	19.29	16.79	14.29	11.79	9.29	6.79	4.29	1.79	(0.71)	(3.21)
	**45,000**	19.57	17.35	15.13	12.90	10.68	8.46	6.24	4.01	1.79	(0.43)
	**50,000**	19.79	17.79	15.79	13.79	11.79	9.79	7.79	5.79	3.79	1.79

Costs savings (or losses) per patient depending on the number of admissions per year and the total fixed costs per year for a virtual reality (VR) therapy program. The value in each cell is the projected cost savings per patient; the value in parentheses indicates a net loss per patient. As an example, for a hospital with 20,000 admissions per year, a VR program that has a total of $300,000 in fixed costs per year would be associated with $6.79 in cost-savings per patient. This table assumes the base-case variables, probabilities and costs described in Table 3. Contact the authors to obtain model results under alternative assumptions.

The bold values are the number of "admissions per year" to the hospital

**Table 3:** Base-case probabilities, costs, and other variables and ranges tested

Variable	Base-case probability (%)	Range tested (%)
VR patient eligibility and acceptance^a,^^21^	19.3	10–50
VR minor adverse effect^21,22^	2.5	0–5
VR major adverse effect^43^	0.025	0–0.5
ORADE rate^30,31^	2.4	1.8–13.6
Opioid utilization^26-29^	65	10–75

Variable	Base-case cost ($)	Range tested ($)
VR^b,^^40^	246,090	50,000–1,000,000
Minor side effect^41,42,44^	28	0–56
Major side effect^45^	4915	500–10,000
Opioid utilization per patient^26-29,44,47,-49^	36	0–100
ORADE^3,4,27,28,30,50^	3,457	804–6870
Last day of hospitalization^51^	584	250–1100

Variable	Base-case multiplier (%)	Range tested (%)
Reduction in opioid utilization associated with use of VR^c^	24	10–80
Reduction in LOS associated with use of VR (for patients without VR adverse effects)^c^	20	0–100
Reduction in LOS associated with use of VR (for patients with minor VR adverse effects)^c^	0	0–100

*VR* virtual reality, *ORADE* opioid-related adverse drug event, *LOS* length of stay

^a^Assumes patient acceptance rate of 50% and inclusion of patients on respiratory and contact isolation

^b^Based on pricing strategy of AppliedVR. See Table 4 for more detailed description of VR cost calculation

^c^No data supporting base-case estimate. The estimate is an assumption. The base-case multiplier represents the marginal cost reduction of the final day of hospitalization associated with VR use

## Discussion

In light of the need for alternative non-pharmacologic pain management and the growing use of VR in healthcare and medicine, we assessed the economic implications of implementing an inpatient VR therapy program for acute pain management. Our results demonstrate that VR therapy may be cost-saving for a hospital system and delineate cost and effectiveness thresholds for VR to remain cost-saving.

In our analysis, decreased hospital length of stay associated with the utilization of VR therapy was the most important factor in determining whether VR therapy is cost-saving. In fact, in our base-case, if hospital length of stay was not reduced, then the implementation of a VR therapy program was not cost-saving even when considering opioid utilization reduction and increased HCAHPS scores. While there is a growing literature around the use of VR in healthcare settings, we are not aware of any prior work that has evaluated whether use of VR in the inpatient setting can decrease length of stay. This is an area worthy of further investigation, and it is the subject of our future research.

In isolation, direct costs savings from reductions in opioid utilization were not large enough to make up for the costs associated with implementing a VR therapy program. However, our analysis only included costs of medication utilization and complications during the initial hospital stay. It was beyond the scope of the model to account for opioid addiction potential, complications after discharge, or quality of life; it is possible that accounting for these factors may show a reduction in costs, particularly for integrated health systems and health maintenance organizations.

Similarly, in our model, raising HCAHPS scores did not create enough value to overcome the costs of VR therapy implementation. Among the variables examined in the tornado analysis, raising HCHAPS scores had the smallest effect on the cost savings potential of an inpatient VR therapy program. HCAHPS scores represent 25% of the VBP, which make up only 2% of total Medicare reimbursements. Additionally, VR as an intervention only affects two of the nine dimensions patients are surveyed about–pain control and the overall rating of the hospital. Therefore, in our model there was a relatively small and limited amount of potential increases in Medicare reimbursement related to improving HCAHPS scores. Furthermore, only a minority of patients in our model received VR therapy, so although their HCAHPS scores rose significantly, in total, scores for the entire hospital only rose marginally. It therefore appears difficult to raise HCAHPS scores from a single intervention that is not extensively utilized and only affects a minority of the surveyed HCAHPS questions.

There are limitations to our study. We based our analysis on many factors that have not been previously studied and for which there are no data. To account for this limitation, we made “best guess” conservative estimations for the base-case, and then subsequently performed extensive one-way sensitivity analyses. Moreover, this work is primarily hypothesis-generating and our primary objective was to determine scenarios in which VR is cost-saving. For example, we found that the strongest driver of VR-associated cost-savings is length of stay reduction. Another limitation is that VR therapy may have other benefits to a hospital system that we did not include in our analysis. It is possible that hospitals that use VR may be viewed by the public and media as technologically advanced and innovative; this may lead to increased revenue for the institution by attracting more patients and increasing market share. Another limitation in our model is that we cannot eliminate selection bias for patients electing to participate in VR, as those choosing to participate may have better outcomes due to predetermined biases about the technology. Lastly, our estimate on the cost of VR implementation was based on a pricing strategy that may change as the VR industry continues to grow and mature.

In summary, our analysis demonstrates the combinations of cost and effectiveness necessary for inpatient VR therapy pain management programs to be cost-saving. Investigators, industry, and healthcare executives can use these data to guide them as they develop, price, and ultimately decide whether to employ therapeutic VR interventions in the acute inpatient setting. This study also brings to light important questions future research should address regarding VR therapy utilization in healthcare. Despite a rich literature regarding pain VR therapy, there are no randomized controlled trials demonstrating the actual efficacy of VR therapy on pain reduction, decreased opioid utilization, or decreased hospital length of stay.

VR therapy has exciting and significant potential applications in medicine, but future research should exam whether VR provides value beyond entertainment to patients, providers, and payers.

## Methods

### Model overview

We used decision analysis software (TreeAge Pro, version 2016, TreeAge Software, Inc., Williamstown, MA) to evaluate the projected cost savings and budget impact of an inpatient VR program vs. usual care on direct hospital outlays. We supported the model with probability estimates from the literature and cost estimates using a hospital payer perspective. We performed sensitivity analyses to estimate the program’s health economic performance in hospitals of varying size and staffing, recognizing that base-case results may not generalize to all settings. In the sections, below, we describe the health economic analyses, competing management strategies, cost accounting, clinical probability estimates, and sensitivity analyses.

### Health economic model

**Fig. 2:**
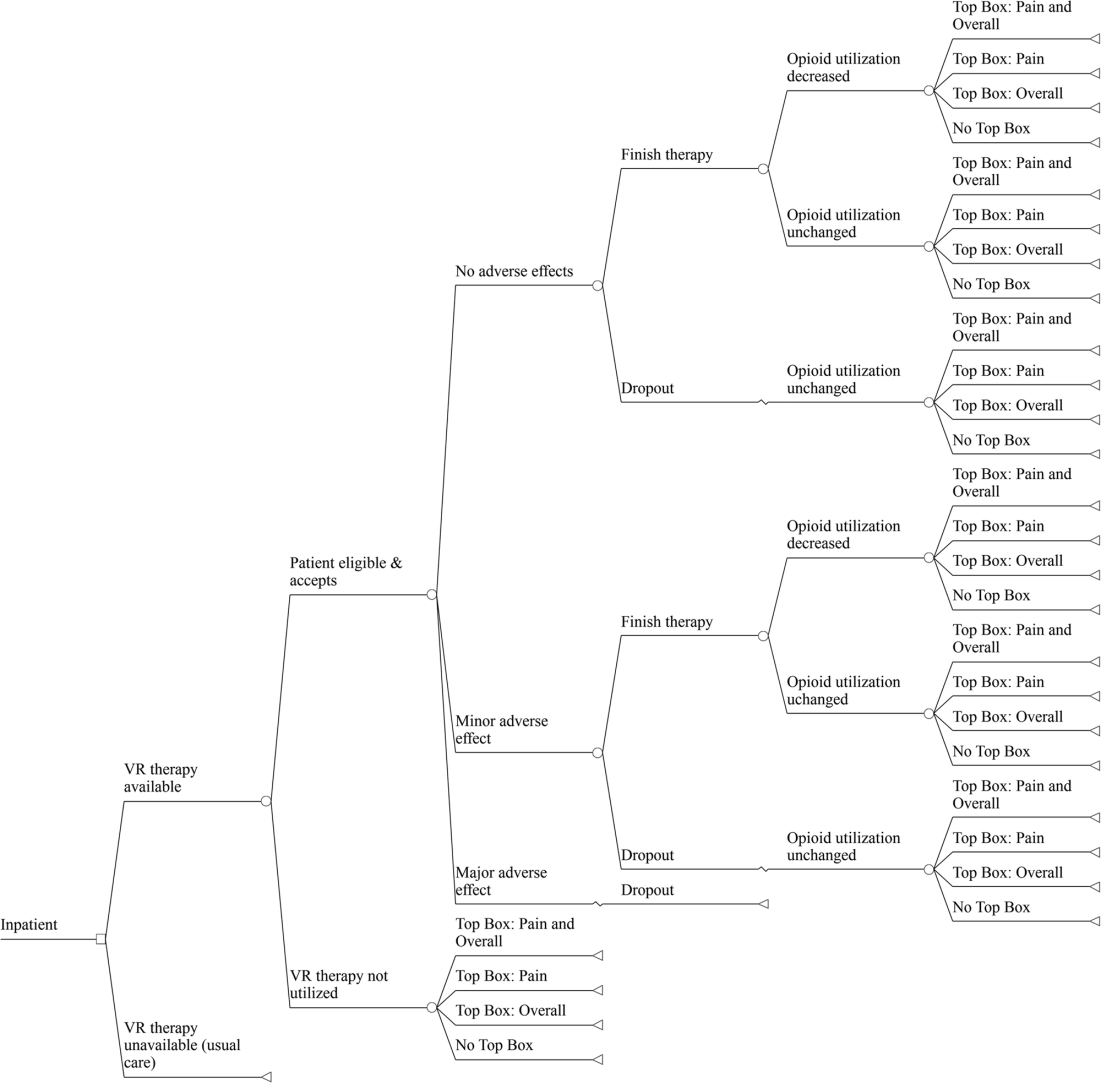
Truncated Decision Model. In the base-case, a general hospital inpatient underwent 1 of 2 competing strategies: an inpatient stay with available VR therapy or an inpatient stay without available VR therapy (status quo). In the setting of available VR therapy, the patient would then have to be eligible for and accept VR therapy, and subsequently experience either no adverse effects, minor adverse effects, or a major adverse effect. The patient chooses whether to complete the full course of VR therapy with resultant changes in opioid utilization, satisfaction, and length of stay. “Top Box” refers to the patient selecting the best possible answers to the HCAHPS survey questions related to the Pain and/or Overall domains. VR, virtual reality; HCAHPS, Hospital Consumer Assessment of Healthcare Providers and Systems

Figure 2 displays the truncated decision model evaluating the health economic outcomes of implementing a hospital-based VR program vs. usual care for pain management. The model simulates the health economic effects of the competing management strategies over a one-year time horizon.

### Competing strategies

Patients in the VR strategy are evaluated for eligibility and, if appropriate, managed by VR therapy as an adjuvant to usual pain treatment. The model assumes that patient adoption of VR depends on factors described in previous research^21,22^: the presence of clinically significant pain, defined as a score ≥3 on the 0 to 10-point visual analogue scale and the absence of exclusions for using VR (i.e., vertigo, epilepsy, recent stroke, nausea, vomiting, facial injury, severe frailty). Based on these factors, we estimated that 38.6% of hospital inpatients are eligible for VR therapy. Moreover, based on VR uptake in our ongoing trials in the inpatient setting (data not yet published), we assumed that 50% of eligible patients accept VR as an adjuvant pain management therapy program. Among these patients eligible and willing to use VR, treatment is administered using visualizations designed for pain management, such as those tested in previous research.^21,22^ VR headsets are made available to patients throughout their hospital stay, as tolerated. In contrast, patients in the usual care arm are not exposed to VR. In both arms, patients may receive opioids or other pain therapies. However, in the VR strategy we assumed that VR could reduce pain beyond usual care based on published controlled data.^22,25^ Additionally, we modeled the potential of VR to reduce opioid usage vs. usual care.

### Clinical probability estimates

#### Virtual reality estimates

In order to inform our base-case probability estimates for VR, we performed a search of published reports from MEDLINE to identify relevant English-language publications from January 1990 to December 2016 concerning use of VR for pain. Because all of our base-case estimates are unlikely to be precisely reproduced in varying populations, we varied each estimate over a wide range in sensitivity analyses, as described below. Table 2 displays the base-case estimates and ranges for probabilities employed in the model.

#### Inpatient opioid utilization estimates

We estimated status quo probabilities for inpatient opioid utilization and opioid-related adverse drug events (ORADE) using weighted averages of data from pertinent studies in MEDLINE (Table 2).^26–31^

#### HCAHPS and VBP reimbursements

We estimated the potential effects of inpatient VR therapy on increasing or decreasing HCAHPS scores and ultimate VBP reimbursements. We used CMS data from fiscal year (FY) 2016 in our calculations.^32–38^ Offering VR therapy for pain could potentially influence patients’ answers in two domains of the HCAHPS survey: pain control and the overall rating of the hospital.^39^ The hospital earns a score from 0 to 10 for each of these domains, depending on the hospital’s performance or improvement during specified time periods. These scores are based on the percentage of patients surveyed that select the best possible answer for each question. For FY 2016 (based on a performance period of January 1, 2014 to December 31, 2014 and a baseline period of January 1, 2012 to December 31, 2012), the average scores for all hospitals participating in the VBP program were 2.35 and 2.62 for the pain control and overall rating dimensions, respectively.^38^ Based on the calculation CMS uses and assuming these domain scores correlate directly to performance scores, for FY 2016, we estimated that on average, 72% of patients selected the best possible answer for the pain control domain, and 73% selected the best possible answer for the overall rating domain. We used these status quo probabilities to estimate similar probabilities in our decision tree model. The full list of probabilities are described in Supplementary Tables 1, 2, and 3.

### Cost estimates

We analyzed direct and indirect costs related to VR therapy implementation, cost savings from possible opioid utilization reduction and reduction in hospital LOS, and potential increases in HCAHPS-related reimbursements. Table 2 includes all cost estimates.

#### VR associated costs

Table 4 summarizes our estimates of the direct fixed and variable costs of instituting an inpatient VR program. Fixed costs included VR annual licenses and salary for a “virtualist” technician responsible for optimizing VR therapeutic delivery to patients. The VR annual license cost estimate was based on the pricing strategy of AppliedVR (www.appliedvr.net), a company that provides bundled VR services with licenses that include a VR headset, subscription to VR software content, and unlimited training and technical support. In the base case model, the hospital purchases thirty licenses to this VR service. For the virtualist clinician, we estimated a yearly salary of $47,030 based on compensation for medical technicians in similar occupations.^40^ Two virtualists were hired in the base case. Variable costs included disinfectant wipes, bouffant hats, foam liners, and headphones, all of which were required for headset sanitization and infection prevention in published research.^21^ The fixed costs in our model totaled to $246,090 per year and the variable costs were $3.39 per patient.

**Table 4:** VR fixed and variable costs

	Cost ($)
VR fixed costs	
VR annual license^a,b^	3500 per year
Virtualist^c,^^40^	47,030 per year
VR variable costs	
Disinfectant wipes^55^	0.16
Bouffant hat^56^	0.23
Foam liner and headphones^b^	3

*VR* virtual reality

^a^Thirty licenses were hypothetically purchased for the base-case. Each license includes a VR headset, subscription to VR software (content), and unlimited training and technical support

^b^Based on pricing strategy of AppliedVR

^c^Two virtualists were hypothetically hired for the base-case

#### VR adverse effects

We estimated the costs of minor adverse effects, including motion sickness, nausea, dizziness, headache, eyestrain, and anxiety as summarized in Supplementary Table 4.^9,41–44^ The average cost of a minor adverse effect totaled to $28. We estimated the cost of a rare major adverse effect by utilizing the median cost of a seizure hospitalization of $4915^45^ (adjusted to 2016 dollars from $4829 in 2013).^46^

#### Inpatient opioid utilization

We calculated weighted averages of data from pertinent studies in MEDLINE and estimated status quo inpatient opioid utilization to be a morphine equivalent dose (MED) of 123 mg.^26–29,47,48^ Using the average wholesale price (AWP) of common opioids^44^ and an online opioid dose calculator to convert doses of opioids to MEDs,^49^ we estimated a mean cost of $0.29 per MED and $36 per hospitalization. Specific opioid pricing and conversions are described in Supplementary Table 5.

Costs associated with opioid utilization also include those related to ORADEs. Estimates of the cost of an ORADE (adjusted to 2016 dollars)^46^ range from $804 to $6870,^3,4,27,28,30,50^ which we averaged to $3457.

#### Reduced length of stay

We sought to estimate the potential savings from a decreased hospital LOS as a result of VR therapy. Given prior research demonstrating that the majority of costs associated with a hospital admission are fixed and incurred early in the admission, we focused on end-of-stay variable costs to estimate cost savings associated with a reduction in LOS.^51–54^ Taheri et al. in 2000 estimated the variable costs of the final day of a general hospitalization to be $420^51^ (adjusted to 2016 dollars, $584). Any potential cost-savings from a VR-related LOS decrease were based on this figure. For the base-case, patients who completed VR and did not experience side effects were assumed to, on average, have a 20% reduction in costs for the final day of hospitalization; we varied this estimate over a wide range in sensitivity analysis. Further, we assumed that patients who completed VR therapy but had a minor side effect achieved no reduction in costs for the final day of hospitalization; this estimate was also varied in sensitivity analysis. We assumed that patients who did not experience VR had no change in the marginal costs associated with the last day of hospitalization.

#### HCAHPS and hospital VBP reimbursements

Patient satisfaction as measured by the HCAHPS surveys impacts CMS Hospital VBP reimbursements. We therefore analyzed the possible effects of offering VR therapy for inpatients on overall HCAHPS scores and reimbursement.

We utilized the CMS VBP calculation to estimate the effects of varying the pain control and overall hospital rating dimension scores from 0 to 10 on Medicare reimbursements for FY 2016.^32–34,36,37^ We used a CMS estimate of the average hospital base operating diagnosis-related group (DRG) payment for FY 2016 of $28,162,066^34^ and held all other variables in the VBP calculation constant at the national averages. Varying the pain and overall dimension scores from 0 to 10 correlated to a minimum CMS incentive payment of $4.39 per patient and a maximum of $11.70 per patient (maximum difference of $7.31 per patient). The maximum difference from the average national status quo scores (i.e., scoring a 10 for both pain and overall dimensions versus the current national averages of 2.35 and 2.62, respectively) was $5.50 per patient.

We included the HCAHPS calculation in the decision tree by incorporating each patient’s probability of selecting the best possible answer for the questions related to the pain and overall dimensions. Each tree branch was associated with an average probability of selecting the best possible answer, which translated to a dimension score and resultant changes in hospital reimbursement. Supplementary Table 6 and Supplementary Table 7 demonstrate the lookup table used to determine the change in reimbursement per patient resulting from the various probabilities of selecting the best answer for the questions on the HCAHPS survey.

### Sensitivity analyses

Because some of the probabilities included in our analysis are supported by only limited data and/or may not apply to all hospitals, we performed extensive sensitivity analyses for all cost and probability estimates. We first tested the influence of all variables on the model by performing multiple univariate sensitivity analysis (i.e., tornado analysis). Based on the results of the tornado analysis, we subsequently completed one-way sensitivity analyses on the most influential variables to identify thresholds where VR became cost-saving. For all sensitivity analyses, the model accounted for additional costs associated with purchasing more VR licenses and hiring additional staff as the number of patients using VR therapy increased (Supplementary Table 8).

We also conducted Monte Carlo probabilistic sensitivity analysis using 1000 simulations and assumed that all variables followed a triangular distribution with base-case, minimum, and maximum values listed in Table 3.

### Data availability

Data sharing is not applicable to this article as no datasets were generated or analyzed during the current study.

## Electronic supplementary material

Supplementary Tables(DOCX 30 kb)

## Electronic supplementary material

**Supplementary information** accompanies the paper on the *npj Digital Medicine* website (10.1038/s41746-018-0026-4).
