# Author Correction: Economic analysis of implementing virtual reality therapy for pain among hospitalized patients

**DOI:** 10.1038/s41746-018-0044-2

**Published:** 2018-08-23

**Authors:** Sean D. Delshad, Christopher V. Almario, Garth Fuller, Duong Luong, Brennan M. R. Spiegel

**Affiliations:** 1 Cedars-Sinai Center for Outcomes Research and Education (CS-CORE), Los Angeles, CA USA; 2 Department of Medicine, David Geffen School of Medicine at UCLA, Los Angeles, CA USA; 3 Division of Digestive and Liver Diseases, Cedars-Sinai Medical Center, Los Angeles, CA USA; 4 Division of Informatics, Cedars-Sinai Medical Center, Los Angeles, CA USA; 5 Department of Pharmacy, Cedars-Sinai Medical Center, Los Angeles, CA USA; 6 Department of Health Policy and Management, UCLA Fielding School of Public Health, Los Angeles, CA USA

**Keywords:** Health care economics, Pain

**Correction to:** *npj Digital Medicine* 10.1038/s41746-018-0026-4, Published online 13 June 2018

The author would like to update the competing interest statement, which in the original published article stated “B.M.R.S. has received a research grant, administered by his academic institution, from appliedVR (Los Angeles, California)”, to include additional information. The competing interest statement has been changed to “B.M.R.S. received a research grant, administered by his academic institution, and also received $1250 in 2014 for a one-time activity that was unrelated to the published research, both from appliedVR (Los Angeles, California).” This has been corrected in the HTML and PDF version of the Article.

