# Supplementary material for: Economic analysis of implementing virtual reality therapy for pain among hospitalized patients
Source: NPJ Digit Med. 2018 Jun 13;1:22. doi: 10.1038/s41746-018-0026-4 (PMC6550142; doi:10.1038/s41746-018-0026-4)
Supplement: Supplementary file 1 — Supplementary Tables(DOCX 30 kb) [file 41746_2018_26_MOESM1_ESM.docx]

**SUPPLEMENTARY TABLE 1: Base-Case Probabilities for Patients without VR therapy Adverse Effects^1^**

| **Variable** | **Probability (%)** |
| --- | --- |
| Patient completes VR therapy | 90 |
| Patient has decreased opioid utilization | 50 |
| Patient selects the best answer for both HCAHPS survey questions | 90 |
| Patient selects the best answer for the pain HCAHPS survey question | 4 |
| Patient selects the best answer for the overall HCAHPS survey question | 4 |
| Patient does not select best answer for either HCAHPS survey question | 2 |
| Patient has unchanged opioid utilization | 50 |
| Patient selects the best answer for both HCAHPS survey questions | 85 |
| Patient selects the best answer for the pain HCAHPS survey question | 4 |
| Patient selects the best answer for the overall HCAHPS survey question | 8 |
| Patient does not select best answer for either HCAHPS survey question | 3 |
| Patient does not complete VR therapy | 10 |
| Patient has unchanged opioid utilization | 100 |
| Patient selects the best answer for both HCAHPS survey questions | 60 |
| Patient selects the best answer for the pain HCAHPS survey question | 15 |
| Patient selects the best answer for the overall HCAHPS survey question | 21 |
| Patient does not select best answer for either HCAHPS survey question | 4 |

VR, virtual reality; HCAHPS, Hospital Consumer Assessment of Healthcare Providers and Systems.

^1^No data supporting base-case estimates. These estimates are assumptions. Probabilities of indented variables are conditional on the variables less indented.

**SUPPLEMENTARY TABLE 2: Base-Case Probabilities for Patients with VR therapy Minor Adverse Effects^1^**

| **Variable** | **Probability (%)** |
| --- | --- |
| Patient completes VR therapy | 50 |
| Patient has decreased opioid utilization | 40 |
| Patient selects the best answer for both HCAHPS survey questions | 85 |
| Patient selects the best answer for the pain HCAHPS survey question | 6 |
| Patient selects the best answer for the overall HCAHPS survey question | 6 |
| Patient does not select best answer for either HCAHPS survey question | 3 |
| Patient has unchanged opioid utilization | 60 |
| Patient selects the best answer for both HCAHPS survey questions | 70 |
| Patient selects the best answer for the pain HCAHPS survey question | 5 |
| Patient selects the best answer for the overall HCAHPS survey question | 20 |
| Patient does not select best answer for either HCAHPS survey question | 5 |
| Patient does not complete VR therapy | 50 |
| Patient has unchanged opioid utilization | 100 |
| Patient selects the best answer for both HCAHPS survey questions | 52.3 |
| Patient selects the best answer for the pain HCAHPS survey question | 20 |
| Patient selects the best answer for the overall HCAHPS survey question | 20 |
| Patient does not select best answer for either HCAHPS survey question | 7.7 |

VR, virtual reality; HCAHPS, Hospital Consumer Assessment of Healthcare Providers and Systems.

^1^No data supporting base-case estimates. These estimates are assumptions. Probabilities of indented variables are conditional on the variables less indented.

**SUPPLEMENTARY TABLE 3: Base-Case Probabilities for Patients who do not accept or are ineligible for VR Therapy (Status Quo Probabilities)[32-38]**

| **Variable** | **Probability (%)** |
| --- | --- |
| Patient selects the best answer for both HCAHPS survey questions | 52.3 |
| Patient selects the best answer for the pain HCAHPS survey question | 20 |
| Patient selects the best answer for the overall HCAHPS survey question | 20 |
| Patient does not select best answer for either HCAHPS survey question | 7.7 |

VR, virtual reality; HCAHPS, Hospital Consumer Assessment of Healthcare Providers and Systems.

**SUPPLEMENTARY TABLE 4: Estimated Frequency and Costs of Minor Adverse Effects from VR**

| **Adverse Effect** | **Frequency^1^** | **Treatment** | **Cost[44]** | **Weighted Cost** |
| --- | --- | --- | --- | --- |
| Nausea | 75% | Ondansetron 8mg | $37.10 | $27.83 |
| Headache/Eyestrain | 30% | Acetaminophen 1000mg | $0.06 | $0.02 |
|  |  | Ibuprofen 800mg | $0.38 | $0.11 |
| Motion Sickness | 20% | Meclizine 25mg | $0.41 | $0.08 |
| Anxiety | 5% | Diazepam 10mg | $0.30 | $0.02 |
| **Total** | | | | **$28.05** |

^1^No data to support these estimates. These estimates are assumptions.

VR, virtual reality; mg, milligram.

**SUPPLEMENTARY TABLE 5: Estimated Narcotic Costs**

| **Opioid** | **Cost[44]** | **MED[49]** | **Cost/MED** |
| --- | --- | --- | --- |
| Oxycodone 5mg PO | $0.15 | 7.5 | $0.02 |
| Acetaminophen/Oxycodone PO | $1.04 | 7.5 | $0.14 |
| Morphine 15mg PO | $0.41 | 15 | $0.03 |
| Morphine 4mg IV | $2.26 | 12 | $0.19 |
| Hydromorphone 0.5mg IV | $1.60 | 2 | $0.80 |
| Fentanyl 25mcg IV | $1.20 | 12.5 | $0.10 |
| Fentanyl 25mcg transdermal | $21.26 | 60 | $0.35 |
| Meperidine 25mg IV | $1.44 | 2.5 | $0.58 |
| Hydrocodone 5mg/Acetaminophen 325mg PO | $0.54 | 5 | $0.11 |
| Hydrocodone 10mg PO | $6.30 | 10 | $0.63 |
| Tramadol 50mg PO | $0.83 | 10 | $0.08 |
|  |  |  |  |
| Average Cost/MED | | | $0.29 |
| Average Inpatient MED[26-29] | | | $123.36 |
| Average Cost of Inpatient Opioid Utilization | | | $36.22 |

MED, morphine equivalent dose; PO, per os; IV, intravenous; mg, milligram; mcg, microgram.

**SUPPLEMENTARY TABLE 6: Lookup Table for changes in reimbursement per patient associated with the percentage of patients selecting the best possible answer for the questions in the Overall Domain in the HCAHPS survey[32-38]**

| **Patients Selecting the Best Possible Answer for the Overall HCAHPS Survey Question (%)** | **Change in Reimbursement per Patient ($)** |
| --- | --- |
| *Less than 69.32* | -0.96 |
| 69.32 | -0.59 |
| 70.95 | -0.22 |
| 72.58 | 0.14 |
| 74.20 | 0.51 |
| 75.83 | 0.87 |
| 77.46 | 1.24 |
| 79.09 | 1.60 |
| 80.71 | 1.97 |
| 82.34 | 2.33 |
| 83.97 | 2.70 |

HCAHPS, Hospital Consumer Assessment of Healthcare Providers and Systems.

**SUPPLEMENTARY TABLE 7: Lookup table for changes in reimbursement per patient associated with selecting the best possible answer for the questions in the Pain Domain of the HCHAPS survey[32-38]**

| **Patients Selecting the Best Possible Answer for the Pain HCAHPS Survey Question (%)** | **Change in Reimbursement per Patient ($)** |
| --- | --- |
| *Less than 70.18* | -0.86 |
| 70.18 | -0.49 |
| 71.07 | -0.13 |
| 71.95 | 0.24 |
| 72.84 | 0.60 |
| 73.73 | 0.97 |
| 74.61 | 1.33 |
| 75.50 | 1.70 |
| 76.39 | 2.07 |
| 77.27 | 2.43 |
| 78.16 | 2.80 |

HCAHPS, Hospital Consumer Assessment of Healthcare Providers and Systems.

**SUPPLEMENTARY TABLE 8: Base-Case Total Yearly Fixed Costs of VR Program Accounting for Hospital Admissions per Year**

| **Hospital Admissions per Year** | **Number of Licenses Purchased** | **Cost per License per year^1^** | **Virtualists Hired** | **Total Fixed Costs per year ($)** |
| --- | --- | --- | --- | --- |
| 1,000-9,999 | 15 | 3500 | 2 | 146,560 |
| 10,000-19,999 | 30 | 3500 | 3 | 246,090 |
| 20,000-29,999 | 50 | 2500 | 4 | 313,120 |
| 30,000+ | 100 | 2500 | 6 | 532,180 |

VR, virtual reality.

^1^ Based on the pricing strategy of AppliedVR.
